# Next-generation sequencing: what are the needs in routine clinical microbiology? A survey among clinicians involved in infectious diseases practice

**DOI:** 10.3389/fmed.2023.1225408

**Published:** 2023-08-21

**Authors:** Charlotte Michel, Charlotte Martin, Pierre Smeesters, Jean-Christophe Goffard, Thomas Demuyser, Marie Hallin

**Affiliations:** ^1^Department of Microbiology, LHUB-ULB, Université libre de Bruxelles (ULB), Brussels, Belgium; ^2^Department of Infectious Diseases, Saint-Pierre University Hospital, Université libre de Bruxelles (ULB), Brussels, Belgium; ^3^Department of Pediatrics, University Hospital Brussels, Université libre de Bruxelles (ULB), Brussels, Belgium; ^4^Department of Pediatrics, University of Melbourne, Melbourne, VIC, Australia; ^5^Department of Internal Medicine, University Hospital Brussels, Université libre de Bruxelles (ULB), Brussels, Belgium; ^6^Department of Microbiology and Infection Control, Vrije Universiteit Brussel (VUB), Universitair Ziekenhuis Brussel (UZ Brussel), Brussels, Belgium; ^7^Centre for Environmental Health and Occupational Health, School of Public Health, University Hospital Brussels, Université libre de Bruxelles (ULB), Brussels, Belgium

**Keywords:** Next-Generation Sequencing (NGS), clinical microbiological diagnosis, questionnaire, survey, microbiological diagnosis

## Abstract

**Background:**

The translation of Next-Generation Sequencing (NGS) from research to clinical microbiology is increasing rapidly, but its integration into routine clinical care struggles to catch-up. A challenge for clinical laboratories is that the substantial investments made in the required technologies and resources must meet both current and forthcoming needs.

**Methods:**

To get a clinical perspective of these needs, we have sent a survey to infectious diseases clinicians of five hospitals, covering the following topics: NGS knowledge, expected syndromes and patients foreseen to benefit from NGS, and expected impact on antimicrobial prescription.

**Results:**

According to clinicians, benefits of NGS are mostly expected in neurological and respiratory infections diagnostics.

**Conclusion:**

A better dialog between microbiologists and clinicians about hopes and limits of NGS in microbiology may help identifying key investments needed for clinical laboratories, today and tomorrow.

## Background

The implementation of Next-Generation Sequencing (NGS) as a routine diagnostic tool is one of the major current challenges for clinical microbiology laboratories (CMLs). Its use is now firmly established in clinical pathology, genetic and oncology diagnosis (1, 2). However, the use of NGS as a diagnostic tool in a routine CML remains scarce (3). In human microbiology, NGS use is indeed mostly limited to academic or reference laboratories. Most published NGS applications in microbiology relate to research studies, applying either metagenomics (mNGS) to explore microbiota or whole genome NGS to characterize specific microorganisms or analyze molecular epidemiology (2, 4). Molecular epidemiological studies have certainly already improved several public health domains such as outbreak management, guidance of vaccination strategies and antibiotic stewardship. The current consensus is that NGS in microbiology could be more broadly used as it has the potential to improve the accuracy of infection diagnostics and to guide tailored treatment as a single, culture independent, all-in-one diagnostic tool that outperforms current time- and labor-intensive conventional methods.

The current lack of standardized protocols or tools in this constantly evolving field is a major obstacle: CMLs that are willing to take the leap have a plethora of decisions to make regarding technologies (sequencing by synthesis or single molecule sequencing), operational models (in-house vs. outsourcing), infrastructure (sequencing platforms, bioinformatics tools, hardware), human resources and expertise (3–5). To guide these choices, we proposed a questionnaire to clinicians involved in infectious diseases (ID), aiming at understanding their needs and better identifying where to prioritize efforts in our diagnostic laboratory. Indeed, when it comes to diagnostic tools, the perception of needs can be very different between the end-users that are the field clinicians and the service providers that are microbiologists. Thus, we interrogated the clinicians’ expectations regarding the potential added value of NGS for their routine clinical care in infectious diseases.

## Methods

The “Laboratoire Hospitalier Universitaire de Bruxelles – Universitair Laboratorium Brussel” (LHUB-ULB) is a merged clinical laboratory serving five university hospitals located in Brussels, Belgium, representing close to 3,000 beds (Supplementary Figure 1). Between January and August 2019, a survey using Google Forms was sent to the clinicians involved in ID [adult ID specialists, intensive care specialists (IC) and ID pediatricians (PID)]. The questionnaire contained 14 questions exploring 3 main topics: (1) background questions such as clinical practice and experience of the participant, level of knowledge regarding NGS (*n* = 3), (2) syndromes (both acute and chronic) and samples for which the diagnostic arsenal could benefit from NGS (*n* = 4) with a special focus on neutropenic patients (*n* = 5), and (3) expected impact on antimicrobial prescription (*n* = 1). The questions (Q) were mainly of a multiple choice design with a set of pre-defined answers, but also included qualitative open questions with entry of free text (Supplementary Annex 1).

The survey form was first sent for pre-test reviewing to an ID clinician, to ensure clarity of the questions and user-friendliness of the digital form. All seniors (specialists) affected to ID, IC and PID of each hospital were solicited individually by email, and were asked to solicit the juniors (in specialization) working with them at the time. An introductive paragraph explained the objective and design of the study and guaranteed the anonymised results of the survey. No incentive was offered.

Results were analyzed after the first round of answers and, applying a modified Delphi’s method (5), gathered into a new questionnaire form. Each respondent received a personalized second survey to confront his/her answers to the global results and optionally modify them.

## Results

Forty-four clinicians received the invitation. Answer rate was 55% (*n* = 24): 62.5% ID, 25% IC and 12.5% PID; 17% were junior and 83% senior doctors. The clinicians were involved in diverse ID areas including travel, HIV and tuberculosis, oncology, transplantation, IC and pediatrics. Fourteen participants (58%) answered the second round; eight (57%) of them did not modify their answers. Only three questions (Q4, Q6, Q7) regarding syndromes and samples that remain without diagnostic were modified by 3 respondents each (Supplementary Table 1).

The participant’s knowledge of NGS (Q3) was using a scale from 0 to 4 (none to very well): 25% rated 0; 54.2% rated 1; 8.3% rated 2; 12.5% rated 3 and none rated 4.

**Figure 1:**
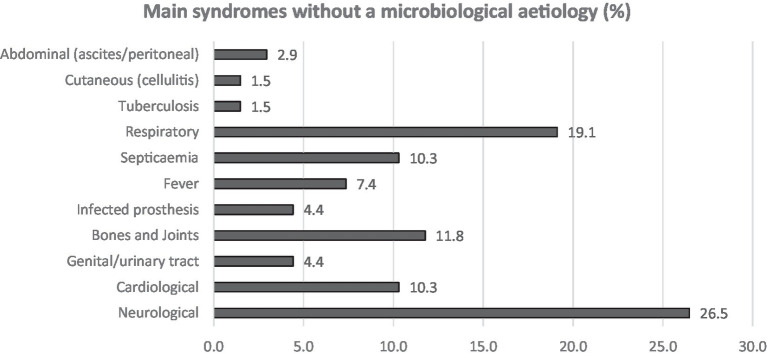
Answers to Q4. Participants were asked to answer up to three syndromes (open fields).

**Figure 2:**
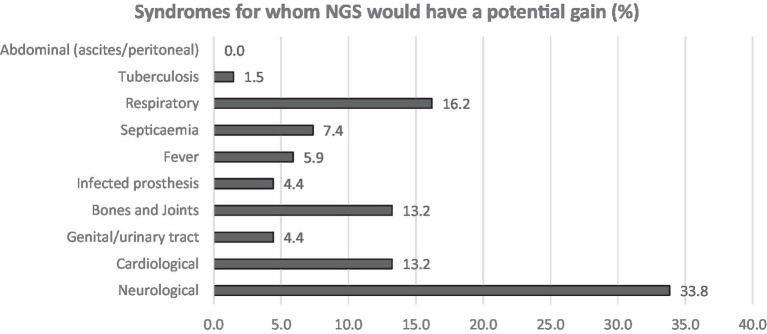
Answers to Q5. Participants were asked to answer up to three syndromes (open fields).

Both Q4 and Q5 had up to three open answer fields. All but five participants (*n* = 19), did fill all the 3 available fields, indicating that our clinicians consider that a wide panel of syndromes and samples remain too often negative even if the suspicion of infection is strong (Supplementary Table 2 and Figures 1, 2).

**Figure 3:**
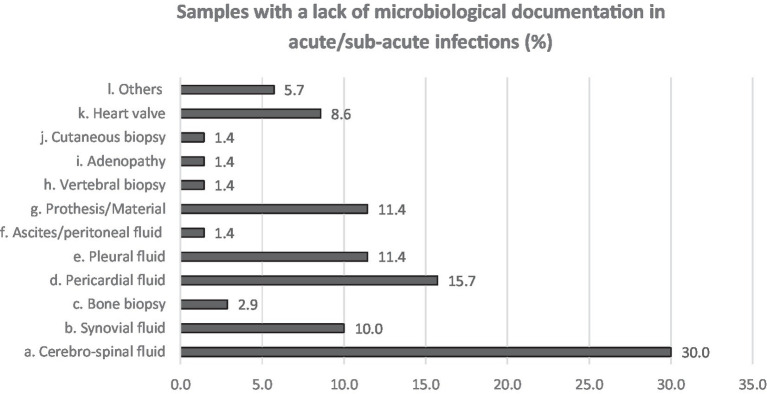
Answers to Q6. Participants were asked to choose up to 3 answers and among a multiple choice list.

**Figure 4:**
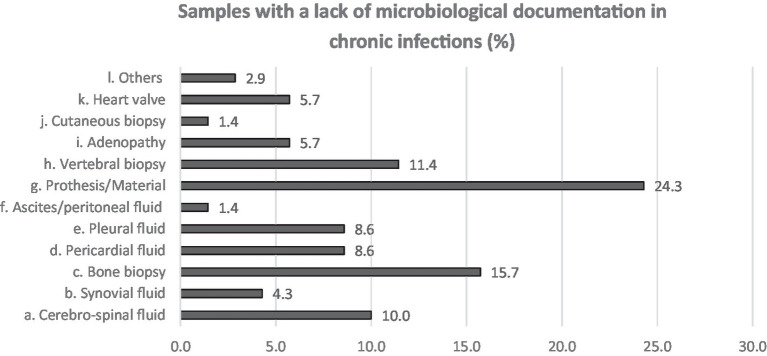
Answers to Q7. Participants were asked to choose up to 3 answers and among a multiple choice list.

Neurological followed by respiratory syndromes and specimens were the most claimed clinical areas (16.2 and 26.5%, respectively) for which participants estimated that the use of NGS could improve the clinical diagnosis, shortly followed by cardiologic and bone and joint infections (10.3 and 11.8% in acute and both 13.2% in chronical infections). From acute infections, cerebrospinal fluid (CSF), pericardial, pleural fluid and prosthetic material were reportedly the sample types that most often lack microbiological documentation and can as such benefit from NGS (Figure 3). For chronic infections, prosthetic material and bone (including vertebral biopsies) were the most frequent selected sample types (Figure 4).

According to 83.3% of participants, the lack of identification of a causative pathogen leads to an empirical treatment management (Q8), meaning that in the majority of cases antibiotics are blindly administrated, and to a lesser extend corticoids (40%) and/or antivirals (25%). But some participants (16.7%) declared to be reluctant to give an answer to this particular question, mostly because the decision is also balanced by their clinical suspicion which is specific of the clinical history of each patient.

All participants reported to treat neutropenic patients on a daily basis (Q10). According to 83.3% of them, a microbiological etiology for these patients’ infections is difficult to obtain. For 70.8% of the participants, this lack of microbiological diagnosis is clearly more frequent in neutropenic patients and this is -for 45.8% of participants- linked to the lack of sensitivity of routine diagnostic tools.

All the participants agreed on the necessity to improve the microbiological diagnostic arsenal.

## Discussion

The potential application of NGS as a routine diagnostic tool in CML, has been recently reviewed (1, 3, 4, 6, 7). However, most of these publications did not explore the expectations of the end-users that are field clinicians. We present here a small-scale survey aiming at exploring the opinion of a panel of clinicians of diverse ID areas regarding the potential added value of NGS for their routine clinical care. An obvious limitation of our study is the relatively low response rate and the size of our sample. Given that the recruitment was voluntary based, a bias toward more interested clinicians, not fully representative of the entire population, is also possible.

Following this survey of the clinicians’ expectations, it is obvious that an improvement is expected in infectious diseases diagnosis. Since lots of infectious episodes remain undocumented, treatment decisions can be of major concern. Sometimes the symptoms suggest either an immune disorder or an infection and the treatments (corticoids versus antimicrobial) have opposite effects (8).

A first striking observation is that almost 80% of the participants considered that they were not familiar with the technique. Even though the SARS-CoV-2 pandemic has generalized the NGS use for the follow-up of variants of concern and may have improved some peculiar knowledge, a significant gap remains between microbiologists and clinicians. The question of filling up this gap should be addressed, as the adequate use of NGS as a routine diagnostic tool implies an understanding of its benefits and limits as compared to existing techniques as well as an enlightened and accurate interpretation of its results by clinicians.

Based on our results, acute and chronic neurological and respiratory infections, cardiologic and bone and joints infections are the areas where NGS could have the largest impact on diagnostics in the opinion of clinicians. CSF, for instance, was quoted as the sample associated with the highest microbiological documentation failure, which is in accordance with the fact that up to 15–60% of meningitis and 70% of encephalitis remain undocumented (8–10). Unfortunately, the capacity of mNGS for viral detection in CSF remains poor (11–13). A few case studies describe NGS helping documenting infection by rare pathogens (9, 12, 14–16) but a large study on acute meningo-encephalitis showed disappointing results: only 22% of the infections were diagnosed by mNGS compared to 45% by conventional methods (13). In contrast, Hasan et al. found good sensibility and specificity (100%; 95%, respectively) for a method designed to detect DNA pathogens only (11).

Similarly, 16.2% of the participants believe that NGS would be useful for respiratory infections diagnostic, in which a wide variety of microorganisms may be involved, especially in lung transplants, IC patients, or for those with severe respiratory illness (17–20). Here, mNGS has demonstrated its added-value, through a whole microbiota-defining approach. The diversity of the pulmonary microbiota (lowered in patients with a confirmed infection) can be, by itself, a marker of Ventilation-associated Pneumonia (VAP) (18, 21). Additionally, mNGS, by detecting rare or difficult to cultivate pathogens and more co-infections, has the potential to enable a more effective adjustment of the anti-infectious regimen, especially as it can also potentially highlight the presence of resistance genes (22). Unfortunately, this low consistency with culture leads to interpretation difficulties regarding, for instance, the relevance of uncultured bacteria in the pathogenesis of VAP (22).

Regarding ‘clean’ orthopedic procedures such as periprosthetic joint infection (PJI), mNGS showed no superiority to culture when compared to clinical scores (7, 23). mNGS presented limited agreement with culture as it had a higher positivity rate and was usually polymicrobial (23–26), identifying bacterial species considered as infrequently associated with clinical infections, sometimes even in patients that were not suspected of infection. Therefore, the clinical relevance of mNGS results and its role in diagnostic algorithms have yet to be determined (24, 25).

Regarding cardiologic infections, good performances were reported for mNGS on native heart valves (27) but the benefit compared to the current 16S rRNA PCR techniques is not significant yet (28). Like for PJI, the exact significance of polymicrobial results has to be explored (28). NGS has also been studied to detect viral genomes in serum of patients presenting acute myocarditis. The results showed a poor sensitivity as well as the detection of viruses of inconclusive pathogenicity to humans (29).

Finally, in immunocompromised hosts -especially febrile neutropenic patients- the range of pathogens potentially involved is wide, fastidious organisms are frequent and the differentiation from non-infectious fever can be difficult. Therefore, mNGS development is particularly long-awaited in this population. A retrospective study on patients with hematologic malignancies and stem cell transplant recipients with persistent fever showed encouraging results regarding a cell-free DNA NGS protocol on blood samples, which identified in a rapid turnaround time several opportunistic pathogens such as *Nocardia*, *Pneumocystis* or non-tuberculous mycobacteria that were either not in the initial differential diagnosis, or missed by conventional methods, helping thereby the antimicrobial treatment adaptation (30). However, the authors also reported a high false positive rate, linked to the detection of clinically non-significant organisms.

## Conclusion

Although the cohort considered here was small and the evaluation fitted to our own practice, our results highlight clinicians’ agreement that the diagnosis in microbiology should be improved and that NGS is considered as the promising technique to do so, especially for the diagnosis of neurological, cardiologic and respiratory infections. However, while major obstacles to the routine use of NGS in CMLs remain, our study reveals a possible gap between field clinicians’ expectations and the actual performances, technical limitations, and difficulties of interpretation of NGS in these clinical situations. Therefore, efforts to better understand the clinicians’ needs should be undertaken in parallel with the development of routine diagnostic protocols by mNGS. Moreover, when such mNGS protocols are implemented, both structural support for the interpretation of the results and continuing formation programs should be provided by molecular biologists and microbiologists to address clinicians’ knowledge gaps.

## Data availability statement

The original contributions presented in the study are included in the article/Supplementary material, further inquiries can be directed to the corresponding author.

## Ethics statement

Ethical review and approval was not required for the study on human participants in accordance with the local legislation and institutional requirements. Written informed consent from the participants was not required to participate in this study in accordance with the national legislation and the institutional requirements.

## Author contributions

CMi: data production and analysis, literature review, and writing. MH: data analysis, literature review, and writing. CMa, PS, J-CG, and TD: reviewing. All authors contributed to the article and approved the submitted version.

## Conflict of interest

The authors declare that the research was conducted in the absence of any commercial or financial relationships that could be construed as a potential conflict of interest.

## Publisher’s note

All claims expressed in this article are solely those of the authors and do not necessarily represent those of their affiliated organizations, or those of the publisher, the editors and the reviewers. Any product that may be evaluated in this article, or claim that may be made by its manufacturer, is not guaranteed or endorsed by the publisher.
