# Supplementary material for: Next-generation sequencing: what are the needs in routine clinical microbiology? A survey among clinicians involved in infectious diseases practice
Source: Front Med (Lausanne). 2023 Aug 21;10:1225408. doi: 10.3389/fmed.2023.1225408 (PMC10475535; doi:10.3389/fmed.2023.1225408)
Supplement: Supplementary file 2 [file Data_Sheet_1.docx]

Annex 1. Questionnaire form that was sent primarily to the clinicians via Google form application

**QUESTIONNAIRE CONCERNING THE IMPLEMENTATION OF N.G.S. AS A ROUTINE TOOL FOR MICROBIOLOGICAL DIAGNOSTIC**

Name:

First Name:

Grade:

Hospital:

E-mail:

Next Generation Sequencing (NGS) is a technology for molecular biology used in the research field of microbiology for a decade. Its development has permitted to sequence a whole genome of microorganism in a much shorter time than the previous techniques. Nowadays, clinical microbiology department try to implement it as a diagnostic tool. Indeed, numerous infectious episodes remain without documented aetiology and a doubt with an immune disorder can persist. This may be of clinical urgence as therapeutic options are contradictory and be damaging if anti-inflammatory treatment is given during an infections.

In theory, NGS could highlight any pathogen in a clinical sample by blindly amplifying its genome when classic molecular techniques need to target a known genome to amplify genetic material, thus requiring larger volumes of samples.

The LHUB-ULB project to develop the use of metagenomic NGS in infectious diseases and we want to gather the opinion of the clinicians’ collaborators of the 5 university hospital that work with us so we can lead our future clinical researches and point the type of infections that need an improvement first.

Therefore, we propose you this following questionnaire. You may write your answers or encircle your choices

This form will be anonymised before presenting the results, then it will be revised using Delphi’s method which mean that results will be presented to all participants so they can agree with the group tendency by validating their first choices or revise their answers.

1. What is your clinical specialty?
   1. Infectious disease specialist
   2. Hygienist
   3. Intensivist (ICU)
   4. Other: precises: ….
2. In which hospital do you work mostly?
   1. Saint Pierre
   2. Institut Jules Bordet
   3. Erasme
   4. Brugmann
   5. HUDERF (Children’s hospital)
3. Do you know the NGS?
4. Not at all
5. A bit
6. Moderately
7. Well
8. Very well
9. Choose the 3 infectious syndromes that are the most likely to remain without an infectious aetiology after any test in microbiology? Propose up to 3 answers
10. For which syndromes do you believe NGS might bring an improvement in the microbiological diagnostic? Propose up to 3 answers
11. Choose the 3 kinds of samples that are the most likely negative after any microbiological testing, even if the suspicion of an acute or sub-acute infection was very strong and before any treatment. Encircle 3 answers

a. Cerebro-spinal fluid

b. Synovial fluid

c. Bone biopsy

d. Pericardial fluid

e. Pleural fluid

f. Ascites/peritoneal fluid

g. Prothesis/Material

h. Vertebral biopsy

i. Adenopathy

j. Cutaneous biopsy

k. Heart valve

l. Others: precise: …

1. Choose the 3 kinds of samples that are the most likely negative after any microbiological testing, even if the suspicion of chronic infection (and possibly after lines of treatment) is very strong. Encircle 3 answers

a. Cerebro-spinal fluid

b. Synovial fluid

c. Bone biopsy

d. Pericardial fluid

e. Pleural fluid

f. Ascites/peritoneal fluid

g. Prothesis/Material

h. Vertebral biopsy

i. Adenopathy

j. Cutaneous biopsy

k. Heart valve

l. Others: precise: …

1. Does the absence of microbial aetiology lead you to treat with an empiric treatment?
   1. by antibiotics: YES – NO
   2. by antiviral: YES – NO
   3. by corticoids: YES – NO

Others: precise: …

1. Are you regularly confronted to neutropenic patients? YES – NO
2. In your day-to-day work, what kind of pathologies cause the neutropenia?
   1. Solid cancer on treatment
   2. Haematologic malignancies
   3. Shock
   4. Immunosuppressive treatment
   5. HIV
   6. Congenital immune deficiency
3. Do you believe that in neutropenic patients the diagnostic of infections is less often documented? YES - NO
4. Do you believe it to be because of a lack of sensitivity of the actual microbiological techniques? YES - NO
5. Do you believe that it is more due to the clinical context (Immunosuppression) than due to the lack of sensitivity of the microbiological techniques? YES - NO
6. Do you believe that the quality of diagnostic in clinical microbiology has to be improved? YES - NO

REMARKS:

**Thank you for your participation and for your expertise in our study.**

Contact is Charlotte Michel, Resident Microbiology +32 2 555 6562, charlotte.michel@lhub-ulb.be.
