# Supplementary figures and images for: Next-generation sequencing: what are the needs in routine clinical microbiology? A survey among clinicians involved in infectious diseases practice
Source: Front Med (Lausanne). 2023 Aug 21;10:1225408. doi: 10.3389/fmed.2023.1225408 (PMC10475535; doi:10.3389/fmed.2023.1225408)

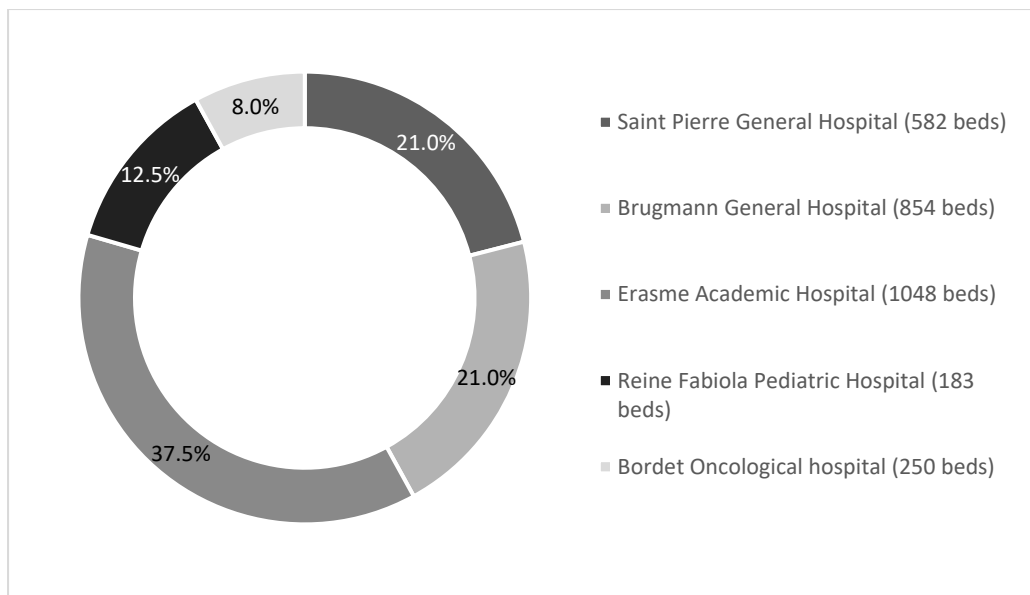

Supplemental Figure 1: Affiliation of participants

Supplement: Supplementary file 3 [file Image_1.pdf]
